# Editorial: Insights in inflammation pharmacology: 2022

**DOI:** 10.3389/fphar.2023.1223761

**Published:** 2023-06-05

**Authors:** Paola Patrignani, Patrizia Ballerini, Per-Johan Jakobsson, Dieter Steinhilber

**Affiliations:** ^1^ Department of Neuroscience, Imaging, and Clinical Science, School of Medicine, and CAST, G. d’Annunzio” University, Chieti, Italy; ^2^ Rheumatology Unit, Department of Medicine, Karolinska Institutet, Karolinska University Hospital, Stockholm, Sweden; ^3^ Institute of Pharmaceutical Chemistry, Goethe University Frankfurt, Frankfurt, Germany; ^4^ Fraunhofer Institute for Translational Medicine and Pharmacology, ITMP and Fraunhofer Cluster of Excellence for Immune-mediated diseases, CIMD, Frankfurt am Main, Germany

**Keywords:** leukotriene, montelukast, lipidomics, glucocorticoids, cholesterol, apolipoprotein A, hepatitis

The Research Topic “Insights in Inflammation Pharmacology-2022” comprises seven papers describing pharmacological intervention options in inflammatory diseases ranging from COVID-19, enteroviral liver infections, and Parkinson’s disease up to rhinosinusitis. The papers address lipid signaling pathways involved in inflammatory reactions, such as cholesterol metabolism and the 5-lipoxygenase pathway. Another article reviews the pharmacological activities of parthenolide.

The paper by Camera et al. reports the effect of the CysLT1 receptor antagonist Montelukast on platelet activation by plasma from COVID-19 patients. Previous data have shown elevated leukotriene levels in bronchoalveolar fluids from intubated COVID-19 patients (Archambault et al., 2021), and targeting the leukotriene pathway has been suggested as a novel strategy to mitigate the hyperinflammatory response in COVID-19 patients (Funk and Ardakani, 2020). In the present study, plasma from COVID-19 patients was added to whole blood, and it was shown that pre-incubation of whole blood with Montelukast (1 µM) inhibits platelet activation induced by COVID-19 patient plasma. It was found that Montelukast prevents the surface expression of tissue factor (TF) and P-selectin, reduces the formation of circulating monocyte- and granulocyte-platelet aggregates and inhibits the release of certain microvesicles. The authors suggest that repurposing montelukast might be an option for the auxiliary treatment for COVID-19 syndrome.

Another paper that addresses leukotriene formation was published by Sud’ina et al. The authors investigated the effects of SkQ1 [10-(6′-plastoquinonyl)decyltriphenylphosphonium bromide] on the leukotriene biosynthesis in human neutrophils. The mitochondria-targeted antioxidant SkQ1 was previously shown to inhibit neutrophil activation by stimuli such as calcium ionophore A23187 or fMLF. The compound accumulates in mitochondria because of the positive charge of the decyltriphenylphosphonium residue and scavenges mitochondrial reactive oxygen species (Antonenko et al., 2008). The present paper shows that SkQ1 at 100 nM strongly inhibits leukotriene formation in human leukocytes in response to various stimuli. Furthermore, SkQ1 effectively inhibits the activation of the MAP kinases p38 and ERK1/2 in neutrophils. Since both kinase cascades are also involved in 5-lipoxygenase activation, the key enzyme of leukotriene formation, the authors suggested that these effects might contribute to the potent inhibitory effects of SkQ1 on leukotriene formation. The authors concluded that developing mitochondria-targeted antioxidants might be a promising strategy to inhibit leukotriene synthesis.

Zhu et al. conducted a study on the transcriptomics and lipidomics profiles in nasal polyps of individuals who responded to glucocorticoid treatment and those who did not. The investigation was carried out before and after treatment. The study aimed to elucidate the mechanisms behind the different responses of patients with chronic rhinosinusitis with nasal polyps to systemic glucocorticoid treatment. The authors performed RNA sequencing, oxidative lipidomics, and differential gene expression analysis in the responder and non-responder groups before and after treatment. It was found that systemic treatment with glucocorticoids leads to anti-inflammatory effects, improves tissue remodeling, restores cilia function, and ameliorates dysregulation of oxylipid mediator pathways, and it was shown that glucocorticoid responders exhibit different transcriptomics signatures than non-responders.

The role of cholesterol metabolism in regulating inflammatory responses is reviewed by Bauer et al. It is widely accepted that cholesterol contributes to atherosclerotic processes, but the role of cholesterol and its biosynthetic precursors in inflammatory processes is still unclear. This mini-review summarizes the current understanding of the inflammation-regulatory properties of cholesterol and several relevant biosynthetic intermediates, particularly emphasizing the different subcellular distributions of the compounds. As an example, the authors describe the effects of cholesterol on NFκB activity regulation and NLRP3 inflammasome activation. Furthermore, the regulatory effects of cholesterol homeostasis on inflammatory processes in the context of SARS-CoV-2 infections are summarized.

Jiang et al. report the effects of apolipoprotein A-I mimetic peptides (ApoAI MP) in a mouse model of Parkinson’s disease. This study aimed to exploit the antioxidant and anti-inflammatory properties of ApoAI MP and explore whether ApoAI MP has therapeutic potential in this disease. It was found that ApoAI MP improved the symptoms of Parkinson’s disease. ApoAI MP increased the antioxidant capacities by increasing the activities of sodium dismutase, catalase, and glutathione peroxidase, which led to decreased malondialdehyde concentration as a marker for lipid peroxidation as well as reduced levels of reactive oxygen species were reported. It was concluded that ApoAI MP improves the behavioral performance of mice in a model of Parkinson’s disease by enhancing antioxidant and anti-inflammatory capacities.

In a review, Wang et al. summarize the therapeutics available for treating fulminant hepatitis caused by enteroviruses in neonates. Hepatitis is one of the most severe and frequent fatal neonatal nonpolio enterovirus infection complications. The disease is frequently caused by Coxsackievirus B (CVB) 1–5 and many echoviruses in neonates and often results in hepatic necrosis followed by disseminated intravascular coagulopathy. The authors report that intravenous immunoglobulin therapy was performed in some clinical trials and summarize the drug repurposing and developing efforts to identify effective treatments.

Zhu et al. summarize the pharmacological profile of parthenolide, a natural compound with characteristics of sesquiterpene lactones which was first isolated from Magnolia grandiflora. It was shown to inhibit the proliferation of cancer cells. Mechanistically, it was found to inhibit DNA biosynthesis in cancer cells and to interfere with NFκB activation. The latter activity inhibits NFκB-mediated expression of genes such as IL-1β, TNF-α, COX-2, iNOS, IL-8, MCP-1, RANTES, ICAM-1, and VCAM-1. Based on this profile, the authors concluded that the compound might be of potential interest for treating arthritis, osteolysis, periodontal disease, and certain types of cancer.
